# Risk factors for COVID-19 patients with cardiac injury: pulmonary ventilation dysfunction and oxygen inhalation insufficiency are not the direct causes

**DOI:** 10.18632/aging.104148

**Published:** 2020-11-23

**Authors:** Sucheng Mu, Wei Wei, Chaoyuan Jin, Ning Pu, Kaihuan Yu, Guorong Gu, Zhe Luo, Chaoyang Tong, Yi Han

**Affiliations:** 1 Emergency Department, Zhongshan Hospital, Fudan University, Shanghai 200032, PR China; 2 Department of General Surgery, Zhongshan Hospital, Fudan University, Shanghai 200032, PR China; 3 Department of Hepatobiliary Surgery, Remin Hospital of Wuhan University, Wuhan 430060, PR China; 4 Department of Critical Care Medicine, Zhongshan Hospital, Fudan University, Shanghai 200032, PR China

**Keywords:** coronavirus disease 2019, COVID-19, cardiac injury, hypertension

## Abstract

Background: Cardiac injury in patients with coronavirus disease 2019 (COVID-19) has been reported in recent studies. However, reports on the risk factors for cardiac injury and their prognostic value are limited.

Results: In total, 15.9% of all cases were defined as cardiac injury in our study. Patients with severe COVID-19 were significantly associated with older age and higher respiratory rates, Sequential Organ Failure Assessment (SOFA) scores, cardiac injury biomarkers and PaO_2_/FiO_2_ ratios. Male patients with chest distress and dyspnea were more likely to have severe disease. Patients with cardiac injury were significantly more likely to have a severe condition and have an outcome of death. However, no significant difference was found in respiratory rates, dyspnea or PaO_2_/FiO_2_ ratio between patients with or without cardiac injury. In the logistic regression model, pre-existing hypertension and higher SOFA score were independent risk factors for patients with COVID-19 developing cardiac injury.

Conclusions: Our study revealed that cardiac injury was an important predictor for patients having a severe or fatal outcome. Patients with pre-existing hypertension and higher SOFA scores upon admission were more likely to develop cardiac injury. Nevertheless, pulmonary ventilation dysfunction and oxygen inhalation insufficiency were not the main causes of cardiac injury in patients with COVID-19.

Methods: A total of 113 confirmed cases were included in our study. Severe patients were defined according to American Thoracic Society guidelines for community-acquired pneumonia. Cardiac injury was defined as a serum cTnI above the 99^th^-percentile of the upper reference limit. Patient characteristics, clinical laboratory data and treatment details were collected and analyzed. The risk factors for patients with and without cardiac injury were analyzed.

## INTRODUCTION

First reported in Wuhan, Hubei Province, China, coronavirus disease 2019 (COVID-19) caused by severe acute respiratory syndrome coronavirus 2 (SARS-CoV-2) has now caused considerable morbidity and mortality in almost all countries [1, 2], with an overall mortality rate of approximately 3.4% [3]. The common clinical features of most COVID-19 patients are fever, cough, sputum production, fatigue, and breathlessness, which are not distinguishable from the symptoms of other respiratory infections [4]. With the progression of this disease to pneumonia, respiratory failure and death often occur in the first week, accompanied by an extreme rise in inflammatory factors such as IL2, IL6, IL10 and TNFα [5]. In the current study, the common complications were acute lung injury, acute respiratory distress syndrome (ARDS), shock, sepsis and kidney injury [4, 6].

Recently, cardiac injury has been reported to accompany SARS-CoV-2 infection, with an incidence ranging from 12% to 19.7% [7, 8]. Manifested as a reduced ejection fraction and elevated troponin I levels, cardiac injury has been reported to play an important role as an independent risk for COVID-19-associated mortality, even more significantly than age, diabetes, chronic pulmonary disease, or history of cardiovascular disease [8, 9]. Multiple factors, including direct viral cardiac damage, pulmonary ventilation dysfunction, pre-existing hypotension, hyperinflammatory responses, ACE2 receptor dysregulation and others, have been considered causes of cardiac injury after coronavirus infection. In this observational retrospective cohort study, we compared the different factors between patients with severe and non-severe COVID-19 and examined the potential risk factors for cardiac injury in patients with COVID-19.

## RESULTS

### Clinical characteristics of the COVID-19 patients

**Figure 1:**
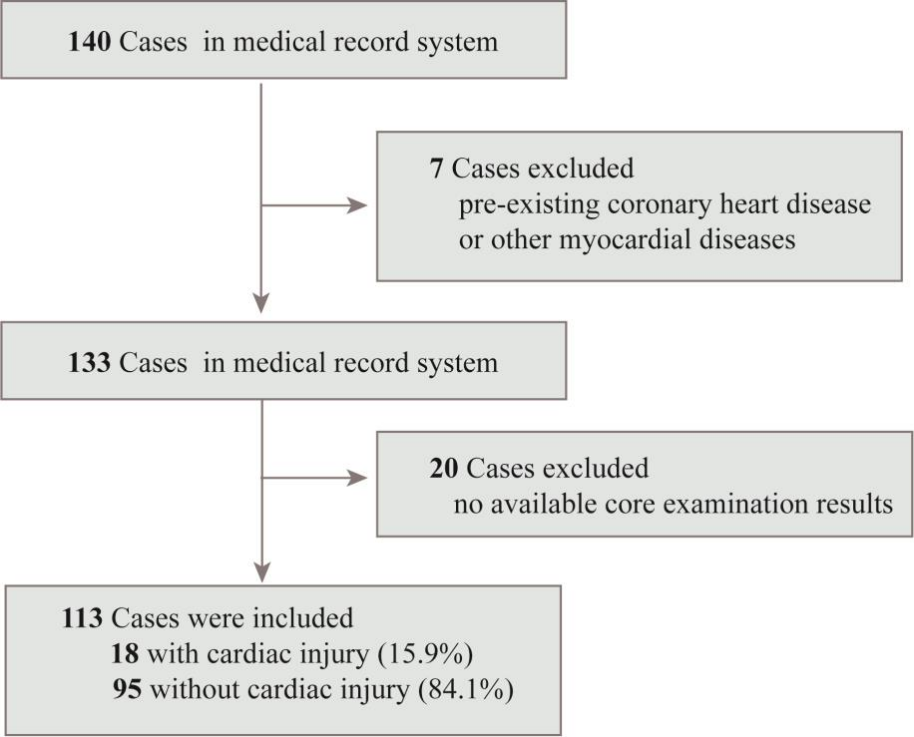
**Flowchart of patients recruitment.**

A total of 140 adult patients with COVID-19 confirmed by SARS-CoV-2 RNA detection in Renmin Hospital of Wuhan University between February 16 and March 21, 2020 were enrolled in this retrospective observational cohort study. After excluding seven patients who were previously diagnosed with coronary heart disease and 20 patients without available basic examinations in their medical records, we included 113 inpatients in the final analysis (Figure 1). The median age of the 113 patients in this study was 63.00 years (IQR 49.50-70.00), ranging from 23 years to 87 years, and 54.9% of them were male. Comorbidities were present in over half of the patients, and the most common comorbidity was hypertension, followed by diabetes (Table 1). Fever (78.8%) was the most common symptom on admission, with a median duration of 10 days (IQR 4.50-15.00). Cough (60.2%) was the second most common symptom, followed by myalgia or fatigue (43.4%). Among all 113 patients, lymphocytopenia occurred in 63 (55.8%) patients, and 18 (15.9%) patients were confirmed to have cardiac injury upon admission. During the hospital treatment period, 92.9% patients were provided with oxygen supplementation, and 14 patients required invasive mechanical ventilation, of whom 9 (64.3%) died. All patients who died were in the severe group.

**Table 1:** Demographic and characteristics of COVID-19 patients in severe or non-severe groups.

	**All patients (n=113)**	**Non-severe patients (n=60)**	**Severe patients (n=53)**	**P value**
**Characteristics**				
Age, years	63.00 (49.50-70.00)	61.00 (43.00-68.75)	67.00 (57.50-72.50)	**0.001**
Sex				**0.045**
Male	62 (54.9%)	28 (46.7%)	34 (64.2%)	
Female	51 (45.1%)	32 (53.3%)	19 (35.8%)	
**Any comorbidity**				
Hypertension	28 (24.8%)	13 (21.7%)	15 (28.3%)	0.275
Diabetes	15 (13.3%)	10 (16.7%)	5 (9.4%)	0.198
Surgery history	11 (9.7%)	6 (10.0%)	5 (9.4%)	0.587
Others	22 (19.5%)	6 (10.0%)	16 (30.2%)	**0.007**
Signs and symptoms				
Fever	89 (78.8%)	45 (75.0%)	44 (83.0%)	0.210
Time of fever, day	10.00 (4.50-15.00)	11.00 (0.75-16.00)	10.00 (5.00-14.00)	0.502
**Highest temperature, °C**				0.058
< 37.3	32 (28.3%)	19 (31.7%)	13 (24.5%)	
37.3-38.0	27 (23.9%)	18 (30.0%)	9 (17.0%)	
38.1-39.0	42 (37.2%)	19 (31.7%)	23 (43.4%)	
> 39.0	12 (10.6%)	4 (6.7%)	8 (15.1%)	
Respiratory rate	20.00 (19.00-22.00)	20.00 (19.00-20.00)	20.00 (19.00-26.00)	**0.014**
Heart rate	89.55±18.52	87.02±15.14	92.42±21.52	0.131
Systolic pressure, mm Hg	133.87±17.16	134.52±15.65	133.13±18.85	0.671
Cough	68 (60.2%)	39 (65.0%)	29 (54.7%)	0.178
Sputum production	32 (28.3%)	14 (23.3%)	18 (34.0%)	0.149
Myalgia or fatigue	49 (43.4%)	22 (36.7%)	27 (50.9%)	0.090
Headache	4 (3.5%)	2 (3.3%)	2 (3.8%)	0.643
Chest distress	31 (27.4%)	12 (20.0%)	19 (35.8%)	**0.047**
Dyspnea	32 (28.3%)	12 (20.0%)	20 (37.7%)	**0.030**
Diarrhea	13 (11.5%)	6 (10.0%)	7 (13.2%)	0.405
PSI score	68.67±31.51	54.53±25.08	84.68±30.55	**< 0.001**
CURB 65 score	1.00 (0.00-1.00)	0.00 (0.00-1.00)	1.00 (1.00-2.00)	**< 0.001**
APACHE-II score	6.20±4.36	3.77±2.78	8.94±4.20	**< 0.001**
SOFA score	2.00 (1.00-3.00)	1.00 (0.00-1.00)	3.00 (2.00-4.00)	**< 0.001**
**Clinical outcome**				
Length of hospital days	26.05±12,92	19.23±10.61	35.34±9.61	**< 0.001**
Mortality	9 (8.0%)	0 (0.0%)	9 (100%)	**0.001**

Data are median (IQR), mean ± SEM, n (%), or n/N (%). p values were calculated by Mann-Whitney U test, t test, χ2 test, or Fisher's exact test, as appropriate. Abbreviation: PSI score, pneumonia severity index score; CURB 65 score, Confusion/Urea/Respiratory rate/Blood pressure 65; APACHE-II score, Acute Physiology and Chronic Health Evaluation II score; SOFA score, Sequential Organ Failure Assessment score.

χ2 test comparing all subcategories.

### Dysfunction of pulmonary ventilation and cardiac injury in severe patients

By March 21, the mean hospitalization time (±SEM) for patients was 35.34 ± 9.61 days, whereas the mean hospitalization time (±SEM) of the patients with non-severe disease was 19.23 ± 10.61 days. Severe patients showed an older median age of 67.00 (IQR 57.00-72.50) than non-severe patients (61.00, IQR 43.00-70.00) (Table 1). Male patients accounted for 64.2% of the patients with severe disease, which was greater than the proportion of males among non-severe patients (46.7%). Common comorbidities, such as hypertension, diabetes and surgery history, were not significantly different between the two groups. However, other comorbidities, including respiratory diseases, digestive tract diseases, and autoimmune diseases, showed dramatic differences. In addition, higher respiratory rates were shown in severe patients, and symptoms of chest distress and dyspnea were closely associated with a severe outcome in COVID-19 patients. Compared with non-severe patients, severe patients required more nasal tubes (52/53, 98.1% vs 39/60, 65.0%, *P* < 0.001), facial masks (46/53, 86.8% vs 3/60, 5%, *P* < 0.001), high-flow nasal cannula oxygen therapy (HFNC) (32/53, 60.4% vs 2/60, 3.3%, *P* < 0.001), noninvasive ventilation (NIV) (18/53, 34.0% vs 0/60, 0.0%, *P* < 0.001) and intermittent mandatory ventilation (IMV) (14/53, 26.4% vs 0/60, 0%, *P* < 0.001) (Table 2).

**Table 2:** Laboratory indicators of COVID-19 patients in severe or non-severe groups.

	**All patients (n=113)**	**Non-severe patients (n=60)**	**Severe patients (n=53)**	**P value**
Blood cell count, × 10^9^ per L				
White blood cell	6.76±3.30	5.92±2.46	7.75±3.87	**0.007**
Neutrophil	3.89 (2.67-7.00)	3.39 (2.19-4.61)	5.96 (3.33-9.80)	**< 0.001**
Lymphocytes	1.10±0.57	1.42±0.56	0.73±0.27	**< 0.001**
Monocytes	0.46±0.21	0.53±0.20	0.38±0.19	**< 0.001**
Platelet count, × 10^9^ per L	226.71±85.51	242.68±86.50	207.68±81.15	0.110
Red cell count, × 10^9^ per L	4.14±0.59	4.23±0.56	4.04±0.62	**0.038**
Haemoglobin, g/L	128.00 (120.00-137.75)	130.50 (120.00-139.75)	127.50 (11950-137.00)	0.557
Albumin, g/L	37.86±5.96	40.29±5.74	34.86±4.76	**< 0.001**
CRP, mg/L	35.80 (5.00-87.18)	6.50 (2.50-38.70)	88.60 (33.60-152.80)	**< 0.001**
ALT, U/L	24.00 (17.00-42.00)	19.5 0(16.00-28.50)	37.00 (21.00-51.00)	**< 0.001**
TB, mmol/L	12.00 (8.90-15.80)	10.90 (8.20-14.30)	13.30 (10.15-17.58)	**0.021**
Potassium, mmol/L	4.08±0.65	4.13±0.54	4.03±0.77	0.456
Sodium, mmol/L	141.68±5.09	143.61±4.30	139.28±5.01	**< 0.001**
Calcium, mmol/L	2.13 (2.03-2.22)	2.18 (2.13-2.28)	2.05 (1.95-2.12)	**< 0.001**
Creatinine, μmol/L	63.00 (53.00-74.00)	63.00 (53.00-71.5)	63.5 (52.50-63.50)	0.979
IL2, pg/L	3.59 (3.17-3.98)	3.56 (3.17-4.12)	3.67 (3.17-3.98)	0.793
IL4, pg/L	3.20±0.63	3.09±0.70	3.31±0.53	0.278
IL6, pg/L	10.20 (5.77-37.20)	6.49 (4.34-11.94)	16.38 (7.65-70.98)	**< 0.001**
IL10, pg/L	5.85 (4.64-7.60)	5.30 (4.32-6.21)	6.19 (5.24-12.16)	**0.007**
TNF-α, pg/L	3.18 (2.68-5.35)	3.01 (2.59-5.71)	3.21 (2.75-5.59)	0.607
INF-γ, pg/L	3.44 (3.01-5.24)	3.42 (2.82-5.38)	3.44 (3.11-4.29)	0.808
LDH, U/L	313.22±153.57	230.29±81.35	416.42±160.25	**< 0.001**
cTnI, pg/mL	0.006 (0.000-0.010)	0.006 (0.000-0.006)	0.007 (0.006-0.028)	**< 0.001**
BNP, pg/mL	144.50 (51.58-369.08)	79.25 (21.51-165.23)	276.05 (119.03-869.43)	**< 0.001**
CK, U/L	58.00 (40.00-103.00)	51.50 (36.25-71.50)	78.00 (51.50-153.50)	**0.002**
CKMB, U/L	1.13 (0.64-1.72)	0.96 (0.61-1.27)	1.48 (0.90-2.82)	**< 0.001**
D-dimer, mg/L	0.81 (0.36-2.30)	0.43 (0.26-1.14)	2.19 (0.83-8.39)	**< 0.001**
Procalcitonin, ng/mL	0.43 (0.13-3.80)	2.80 (0.12-4.03)	0.30 (0.14-2.95)	0.722

Data are median (IQR), mean±SEM. p values were calculated by Mann-Whitney U test or t test as appropriate. Abbreviation: CRP, C-reactive protein; ALT, alanine aminotransferase; TB, total bilirubin; LDH, lactate dehydrogenase; cTnI, cardiac troponin I;BNP, brain natriuretic peptide; CK, creatine kinase; CKMB, creatine kinase-MB.

With the different ventilation supplements in the two groups, the PaO_2_/FiO_2_ ratio (P/F ratio) showed a significant difference between the severe patients (202.67±116.39 mmHg) and non-severe patients (385.70±155.85), indicating that the function of pulmonary ventilation in COVID-19 patients was an important factor associated with the severity of illness.

The severe patients presented higher neutrophils, lower lymphocytes and lower monocytes in both proportion and numbers than patients with non-severe disease (Table 2). In addition, the levels of albumin, C-reactive protein (CRP), total bilirubin (TB), D-dimer, serum IL6, and IL10 were significantly higher in the severe patients compared with the non-severe patients, whereas the level of sodium and calcium were lower in severe patients. It is worth noting that all the biomarkers related to cardiac injury in the severe patients showed dramatically significant differences from those in the non-severe patients (*P* < 0.001, Table 2), indicating that the severe patients were accompanied with relatively severe cardiac dysfunction.

### Kinetic analysis of serum cardiac injury biomarkers in COVID-19 patients and correlation analysis between cardiac injury biomarkers and other parameters

**Figure 2:**
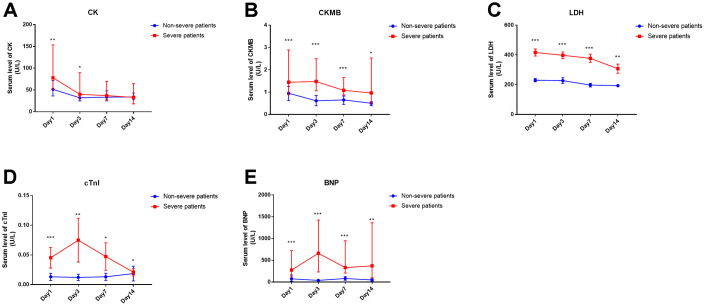
**Time course of cardiac injury biomarkers of COVID-19 patients in severe or non-severe groups.** (**A-E**) serum levels of CK, CKMB, LDH, cTnI and BNP in the two groups. “*” means significant difference between the two groups. *, P < 0.05, **, P < 0.01, ***, P < 0.001.

Serum cardiac injury biomarkers, including CK, CKMB, LDH, cTnI and BNP, were observed as significant predictors for severe patients at the time of hospital admission. Next, we analyzed the kinetic alterations of these five biomarkers over at least 14 days. The significantly increased serum CK in the severe group, compared with that in non-severe patients, was only observed at the onset (within 3 days) but not during the following period of disease progression (Figure 2A). The other four biomarkers were increased significantly in the severe patients, not only on the first day but also during two weeks after admission compared with the non-severe patients (Figure 1B–1E).

**Figure 3:**
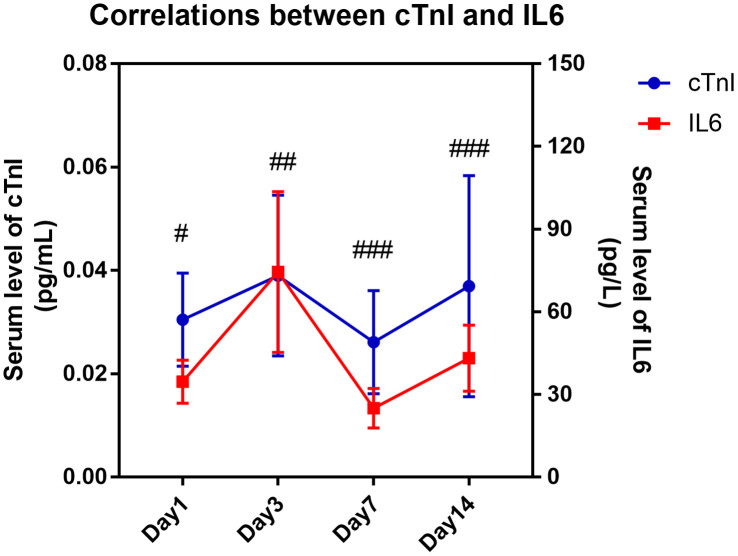
**Kinetic correlation between serum IL6 level with cardiac injury biomarker of COVID-19 patients.** Pearson correlation analysis was performed between serum level of IL6 and serum cTnI on different time points. “#” means serum IL6 level was significantly correlated with serum cTnI level. #, *P* < 0.05, ##, *P* < 0.01, ###, *P* < 0.001.

Due to the high sensitivity and specificity of cTnI in distinguishing cardiac injury and the widespread application of BNP in distinguishing heart failure in clinical diagnoses, we further analyzed the correlation of cTnI or BNP with other parameters, which showed significant differences between the two groups in Tables 1–3. As shown in Table 4, the level of serum cTnI was positively correlated with age (R = 0.208, *P* = 0.027), CRP (R = 0.273, *P* = 0.008), CREA (R = 0.258, *P* = 0.009), serum IL6 level (R = 0.302, *P* = 0.037), pneumonia severity index (PSI) score (R = 0.210, *P* = 0.025), APACHE II score (R = 0.296, *P* = 0.001) and SOFA score (R = 0.323, *P* < 0.001) and negatively correlated with lymphocyte counts (R = -0.245, *P* = 0.012) in all patients. The level of serum BNP was only positively correlated with creatinine (CREA) (R = 0.957, *P* < 0.001), CURB65 score (R = 0.252, *P* = 0.032), APACHEII score (R = 0.241, *P* = 0.042) and SOFA score (R = 0.326, *P* = 0.005). It is worth noting that serum cTnI showed no correlation with CURB65 score (R = 0.163, *P* = 0.085), and serum BNP showed no correlation with PSI score (R = 0.224, *P* = 0.059). Both biomarkers showed no correlation with the P/F ratio (Table 4). In addition, we found that the serum level of IL6 showed a significantly positive correlation with serum cTnI level (*P* = 0.008 on day 3, *P* < 0.001 on day 7 and *P* < 0.001 on day 14), and these two indicators presented similar variation tendency over time (Figure 3).

**Table 3:** Pulmonary ventilation and oxygen content of COVID-19 patients in severe or non-severe groups.

	**All patients (n=113)**	**Non-severe patients (n=60)**	**Severe patients (n=53)**	**P value**
**Oxygenation**				
Nasal tube	91 (80.5%)	39 (65.0%0	52 (98.1%)	**< 0.001**
Facial mask	49 (43.4%)	3 (5.0%)	46 (86.8%)	**< 0.001**
HFNC	34 (30.1%)	2 (3.3%)	32 (60.4%)	**< 0.001**
NIV	18 (15.9%)	0 (0.0%)	18 (34.0%)	**< 0.001**
IMV	14 (12.4%)	0 (0.0%)	14 (26.4%)	**< 0.001**
ECMO	2 (1.8%)	0 (0.0%)	2 (3.8%)	0.218
**Arterial blood gas analysis**				
PH	7.41±0.08	7.41±0.07	7.41±0.08	0.899
PO_2_, mmHg	77.74±33.91	87.56±28.09	75.53±35.02	0.342
PCO_2_, mmHg	40.18±9.63	42.22±7.31	39.73±10.10	0.488
BE, mmol/L	2.43±4.22	2.43±3.80	2.43±4.36	0.998
CHCO_3_^-^st, mmol/L	25.46±4.04	26.76±2.98	25.16±4.22	0.290
FiO_2_,	0.41±0.21	0.24±0.04	0.46±0.21	**< 0.001**
PaO_2_/FiO_2_, mmHg	236.28±142.07	385.70±155.85	202.67±116.39	**< 0.001**

Data are median (IQR), mean±SEM, n (%), or n/N (%). p values were calculated by Mann-Whitney U test, t test, χ2 test, or Fisher's exact test, as appropriate. HFNC, high-flow nasal catheter; NIV, non-invasive ventilation; IMV, invasive mechanical ventilation; ECMO, Extracorporeal Membrane Oxygenation.

**Table 4:** Correlations between cardiac injury biomarkers with the important factors (*P* < 0.05, severe group vs non-severe group).

	**cTnI**		**BNP**	
	Correlation coefficient	*P* value	Correlation coefficient	*P* value
Age	0.208*	**0.027**	0.191	0.108
Sex	0.068	0.471	0.116	0.331
Time of fever	0.014	0.883	0.163	0.171
Hypertension	-0.011	0.912	-0.060	0.619
Diabetes	0.036	0.701	0.301	0.010
Lymphocyte counts	-0.245*	**0.012**	-0.198	0.100
CRP	0.273**	**0.008**	0.047	0.706
ALT	0.191	0.060	-0.074	0.554
CREA	0.258**	**0.009**	0.957**	**< 0.001**
IL2	0.184	0.244	-0.055	0.757
IL4	-0.129	0.416	-0.027	0.878
IL6	0.302*	**0.037**	0.284	0.076
IL10	-0.014	0.932	0.082	0.645
TNF-α	0.025	0.875	0.121	0.496
INF-γ	-0.007	0.969	-0.029	0.883
D-dimer	0.159	0.122	0.016	0.897
P/F ratio	-0.032	0.825	-0.044	0.801
PSI score	0.210*	**0.025**	0.224	0.059
CURB65 score	0.163	0.085	0.252*	**0.032**
APACHEII score	0.296**	**0.001**	0.241*	**0.042**
SOFA score	0.323**	**< 0.001**	0.326**	**0.005**

Correlation analysis between two parameters was performed using Pearson Correlation Coefficient. CRP, C-reactive protein; ALT, alanine aminotransferase; CREA, creatinine; PSI score, pneumonia severity index score; CURB 65 score, Confusion/Urea/Respiratory rate/Blood pressure 65; APACHE-II score, Acute Physiology and Chronic Health Evaluation II score; SOFA score, Sequential Organ Failure Assessment score.

### Dysfunction of pulmonary ventilation in COVID-19 patients was not the main cause of cardiac injury

In total, 113 patients were confirmed to have COVID-19 in our study, 18 patients (15.9%) were defined as having cardiac injury, and 95 patients (84.1%) were defined as having no cardiac injury. As indicated in Table 5, patients with cardiac injury showed significantly higher proportion in severe patients (13/18, 72.2%) than patients without cardiac injury (40/95, 42.1%) (*P* = 0.018). Compared with patients without cardiac injury, patients with cardiac injury were older (median 69.50, IQR 66.50-78.00 years). Moreover, hypertension (*P* = 0.011) as well as a higher heart rate (*P* = 0.011) and systolic pressure (*P* = 0.002) were more common among patients with cardiac injury. No significant difference was observed in respiratory rate or in the proportion of patients with chest distress and dyspnea among patients with cardiac injury, which were dramatically different between severe patients and non-severe patients. The days from fever onset to patient admission showed no significant difference between patients with or without cardiac injury.

**Table 5:** Demographic and characteristics of COVID-19 patients with or without cardiac injury.

	**Patients, No, (%)**			***P* value**
	**All (n=113)**	**Cardiac injury**		
		**Without (n=95)**	**With (n=18)**	
Severity				0.018
Non-severe patients	60 (53.1%)	55 (57.9%)	5 (27.8%)	
Severe patients	53 (46.9%)	40 (42.1%)	13 (72.2%)	
Characteristics				
Age, years	63.00 (49.50-70.00)	62.00 (48.00-69.00)	69.50 (66.50-78.00)	< 0.001
Sex				0.202
Man	62 (54.9%)	45 (47.4%)	6 (33.3%)	
Women	51 (45.1%)	50 (52.5%)	12 (66.7%)	
Any comorbidity				
Hypertension	28 (24.8%)	19 (20.0%)	9 (50.0%)	0.011
Diabetes	15 (13.3%)	12 (12.6%)	3 (16.7%)	0.441
Surgery history	11 (9.7%)	9 (9.5%)	2 (11.1%)	0.553
Others	22 (19.5%)	16 (16.8%)	6 (33.3%)	0.101
Signs and symptoms				
Fever	89 (78.8%)	73 (76.8%)	16 (88.9%)	0.207
Time of fever, day	10.00 (4.50-15.00)	10.00 (4.00-15.00)	10.00 (7.00-14.25)	0.773
Highest temperature, °C				0.716
< 37.3	32 (28.3%)	26 (27.4%)	6 (33.3%)	
37.3-38.0	27 (23.9%)	23 (24.2%)	4 (22.2%)	
38.1-39.0	42 (37.2%)	36 (37.9%)	6 (33.3%)	
> 39.0	12 (10.6%)	10 (10.5%)	2 (11.1%)	
Respiratory rate	20.00 (19.00-22.00)	20.00 (19.00-21.00)	21.50 (19.00-29.00)	0.053
Heart rate, time per s	89.55±18.52	87.86±17.22	98.44±22.83	0.026
Systolic pressure, mm Hg	133.87±17.16	131.75±15.49	145.06±21.35	0.002
Cough	68 (60.2%)	58 (61.1%)	10 (55.6%)	0.426
Sputum production	32 (28.3%)	27 (28.4%)	5 (27.8%)	0.601
Myalgia or fatigue	49 (43.4%)	40 (42.1%)	9 (50.0%)	0.357
Headache	4 (3.5%)	3 (3.2%)	1 (5.6%)	0.506
Chest distress	31 (27.4%)	28 (29.5%)	3 (16.7%)	0.207
Dyspnea	32 (28.3%)	27 (28.4%)	5 (27.8%)	0.601
Diarrhea	13 (11.5%)	10 (10.5%)	3 (16.7%)	0.342
PSI score	68.67±31.51	64.78±32.58	89.22±16.01	**< 0.001**
CURB65 score	0.81±0.79	0.72±0.78	1.33±0.59	**< 0.001**
APACHE-II score	6.20±4.36	5.56±4.31	9.56±2.83	**< 0.001**
SOFA score	2.00 (1.00-3.00)	1.00 (1.00-2.00)	3.00 (2.00-4.00)	**< 0.001**
Clinical outcome				
The length of hospital days	26.05±12,92	25.40±13.34	30.21±9.11	0.100
Mortality	9 (8.0%)	5 (5.3%)	4 (22.2%)	**0.035**

Data are median (IQR), mean±SEM, n (%), or n/N (%). p values were calculated by Mann-Whitney U test, t test, χ2 test, or Fisher's exact test, as appropriate. Abbreviation: PSI score, pneumonia severity index score; CURB 65 score, Confusion/Urea/Respiratory rate/Blood pressure 65; APACHE-II score, Acute Physiology and Chronic Health Evaluation II score; SOFA score, Sequential Organ Failure Assessment score.

χ2 test comparing all subcategories.

The laboratory findings are shown in Table 6. In the patients with cardiac injury, the lymphocyte counts (0.78±0.26, *P* < 0.001), platelet counts (180.17±80.45, *P* = 0.010), serum potassium level (3.7±0.601, *P* = 0.010) and serum calcium level (2.03, IQR 1.97-2.09, *P* = 0.001) were lower than those in patients without cardiac injury. In addition, a higher serum CRP level (63.50, IQR 24.70-154.20, *P* = 0.004), TB level (15.50, IQR 11.85-25.80, *P* = 0.010), IL2 level (3.67, IQR 3.17-3.98, *P* = 0.045), IL6 level (16.38, IQR 7.65-0.98, *P* = 0.003) and D-dimer level (3.20, IQR1.03-15.04, *P* = 0.003) were observed in the cardiac injury group than in the other group. As cardiac injury biomarkers, the serum levels of LDH, BNP, CK and CKMB were all dramatically higher in patients with cardiac injury than in patients without cardiac injury (*P* < 0.001). Although patients with cardiac injury required more oxygen inhalation measures, including facial masks (13/18, 72.2% vs 36/95, 37.9%, *P* = 0.007), HFNC (10/18, 55.6% vs 24/95, 25.3%, *P* = 0.013), NIV (7/13, 38.9% vs 11/95, 11/6%, *P* = 0.009) and IMV (6/18, 33.3% vs 8/95, 8.4%, *P* = 0.010), the indicators of arterial blood gas levels on the second day of admission, including PO_2_, PCO_2_, BE, CHCO_3_^-^st and P/F ratio, showed no significant differences between the two groups (Table 7).

**Table 6:** Laboratory indicators of COVID-19 patients with or without cardiac injury.

	**Patients, No, (%)**			**P value**
	**All (n=113)**	**Cardiac injury**		
		**Without (n=95)**	**With (n=18)**	
White blood cell × 10^9^ per L	6.76±3.30	6.64±3.15	7.41±4.01	0.383
Neutrophil	3.89 (2.67-7.00)	3.81 (2.41-6.36)	5.44 (3.07-8.41)	0.166
Lymphocytes	1.10±0.57	1.17±0.59	0.78±0.26	**< 0.001**
Monocytes	0.46±0.21	0.47±0.22	0.41±0.18	0.253
Platelet count, × 10^9^ per L	226.71±85.51	236.57±83.70	180.17±80.45	**0.010**
Red cell count, × 10^9^ per L	4.14±0.59	4.16±0.61	4.04±0.49	0.130
Haemoglobin, g/L	128.00 (120.00-137.75)	128.00 (119.75-138.25)	130.00 (120.25-138.00)	0.997
Albumin, g/L	37.86±5.96	38.33±5.72	35.49±6.74	0.072
CRP mg/L	35.80 (5.00-87.18)	25.00 (5.00-77.50)	63.50 (24.70-154.20)	**0.004**
ALT, U/L	24.00 (17.00-42.00)	23.00 (17.00-40.25)	32.00 (19.75-71.50)	0.101
TB, mmol/L	12.00 (8.90-15.80)	11.35 (8.50-15.15)	15.50 (11.85-25.80)	**0.010**
Potassium, mmol/L	4.08±0.65	4.16±0.64	3.7±0.601	**0.010**
Sodium, mmol/L	141.68±5.09	142.02±4.82	139.94±6.16	0.124
Calcium, mmol/L	2.13 (2.03-2.22)	2.15 (2.06-2.25)	2.03 (1.97-2.09)	**0.001**
Creatinine, μmol/L	63.00 (53.00-74.00)	62.00 (53.00-74.00)	64.00 (53.50-71.50)	0.616
IL2, pg/L	3.59 (3.17-3.98)	3.56 (3.17-4.12)	3.67 (3.17-3.98)	**0.045**
IL4, pg/L	3.20±0.63	3.09±0.70	3.31±0.53	0.396
IL6, pg/L	10.20 (5.77-37.20)	6.49 (4.34-11.94)	16.38 (7.65-0.98)	**0.003**
IL10, pg/L	5.85 (4.64-7.60)	5.30 (4.32-6.21)	6.19 (5.24-12.16)	0.119
TNF-α, pg/L	3.18 (2.68-5.35)	3.01 (2.59-5.71)	3.21 (2.75-5.59)	0.827
INF-γ, pg/L	3.44 (3.01-5.24)	3.42 (2.82-5.38)	3.44 (3.11-4.29)	0.468
LDH, U/L	313.22±153.57	290.45±142.20	443.73±155.72	**< 0.001**
BNP, pg/mL	144.50 (51.58-369.08)	95.39 (26.66-227.38)	553.10 (299.83-1179.00)	**< 0.001**
CK, U/L	58.00 (40.00-103.00)	53.50 (37.75-86.25)	119.00 (72.00-168.00)	**< 0.001**
CKMB, U/L	1.13 (0.64-1.72)	1.02 (0.62-1.46)	2.31 (1.42-3.53)	**< 0.001**
D-dimer, mg/L	0.81 (0.36-2.30)	0.63 (0.34-1.88)	3.20 (1.03-15.04)	**0.003**
Procalcitonin, ng/mL	0.43 (0.13-3.80)	0.55 (0.13-4.45)	0.39 (0.11-1.20)	0.273

Data are median (IQR), mean±SEM. p values were calculated by Mann-Whitney U test or t test as appropriate. Abbreviation: CRP, C-reactive protein; ALT, alanine aminotransferase; TB, total bilirubin; LDH, lactate dehydrogenase; cTnI, cardiac troponin I; BNP, brain natriuretic peptide; CK, creatine kinase; CKMB, creatine kinase-MB.

**Table 7:** Pulmonary ventilation and oxygen content of COVID-19 patients with or without cardiac injury.

	**Patients, No, (%)**			**P value**
		**Cardiac injury**		
	**All (n=113)**	**Without (n=95)**	**With (n=18)**	
**Oxygenation**				
Nasal tube	91 (80.5)	74 (77.9)	17 (94.4)	0.089
Facial mask	49 (43.4)	36 (37.9)	13 (72.2)	**0.007**
HFNC	34 (30.1)	24 (25.3)	10 (55.6)	**0.013**
NIV	18 (15.9)	11 (11.6)	7 (38.9)	**0.009**
IMV	14 (12.4)	8 (8.4)	6 (33.3)	**0.010**
ECMO	2 (1.8)	1 (1.1)	1 (5.6)	0.294
**Arterial blood gas analysis**				
PH	7.41±0.08	7.39±0.08	7.48±0.04	**0.002**
PO_2_, mmHg	77.74±33.91	77.13±28.75	80.10±51.34	0.864
PCO_2_, mmHg	40.18±9.63	41.21±10.25	36.20±5.37	0.144
BE, mmol/L	2.43±4.22	2.23±4.19	3.21±4.50	0.519
CHCO_3_^-^st, mmol/L	25.46±4.04	24.93±4.08	27.49±3.33	0.074
FiO_2_,	0.41±0.21	0.42±0.20	0.39±0.24	0.714
PaO2/FiO2, mmHg	236.28±142.07	237.82±151.39	230.32±104.03	0.883

Data are median (IQR), mean±SEM, n (%), or n/N (%). p values were calculated by Mann-Whitney U test, t test, χ2 test, or Fisher's exact test, as appropriate. HFNC, high-flow nasal catheter; NIV, non-invasive ventilation; IMV, invasive mechanical ventilation; ECMO, Extracorporeal Membrane Oxygenation.

In patients with cardiac injury, the PSI (89.22±16.01 vs 64.78±32.58, *P* < 0.001), CURB65 (0.72±0.78 vs 1.33±0.59, *P* < 0.001), APACHEII (9.56±2.83 vs 5.56±4.31, *P* < 0.001) and SOFA scores (3.00, IQR 2.00-4.00 vs 1.00, IQR 1.00-2.00, *P* < 0.001) were all higher than in those without cardiac injury. As APACHEII score was calculated based on age and other scores (PSI, SOFA, CURB65) were related to the rest of the laboratory indicators, we chose age, hypertension, PSI, CURB65 score and SOFA score as the five variables for our multivariable logistic regression model. A significant difference was observed in the logistic model with χ^2^(5) = 21.998, P <0.0005. The predictive model was able to classify 86.7% of the cardiac injury patients among patients with COVID-19, with a sensitivity of 33.3% and specificity of 96.8%. Under this hazard regression model, the variables age, CURB65 score and APACHEII score showed no significant difference between groups, and they were not independent risk factors for cardiac injury of patients with COVID-19. Regarding the independent risk factors, patients with pre-existing hypertension were 3.2 times more likely to have cardiac injury than those without hypertension (OR 3.28, 95% CI 1.02-10.61), and the risk of cardiac injury rose 66% with a one-score increase of the SOFA score on the hospital admission day.

## DISCUSSION

In the present study, our findings indicated that pulmonary ventilation dysfunction were not directly associated with cardiac injury in patients with COVID-19. In contrast, patients with pre-existing hypertension and elevated SOFA scores upon admission, which were regarded as independent risk factors in our study, were more likely to progress to cardiac injury.

The elevated biomarker levels in severe patients and cardiac injury patients indicated that the myocardial damage in COVID-19 patients were not random. This factor might be associated with the outcome with the patients. A previous study pointed out that in hospitalized patients with COVID-19, cardiac injury is a common condition in disease progression, and it is tightly associated with a higher risk of in-hospital mortality [8]. The elevated levels of biomarkers, such as BNP and troponin, were regarded as the prominent features in COVID-19 patients and reported to be associated with ICU admission and mortality [6, 9–11]. Cardiac involvement is of great importance in determining the prevalence and prognosis of COVID-19 patients. In our current study, the proportion of cardiac injuries in the severe patients or non-survival patients was significantly higher than that in non-severe patients or survival patients, respectively, which was similar with previous study.

Exaggerated systemic inflammation, lymphocytopenia, hypoxemia and cardiovascular stress might be the hallmarks of severe patients with COVID-19 [10]. In our current study, lymphocytopenia was more common in severe patients in terms of the numbers of lymphocytes and monocytes, as well as in patients with cardiac injury, than in patients without cardiac injury. The inflammatory factor IL6 showed similar alterations and was significantly higher in both patients with severe disease and patients with cardiac damage than in their corresponding comparison groups. Compared with IL6, some other cytokines, such as IL2 and IL10, are elevated in cardiac injury patients and severe patients, respectively, activating the pathways leading to the differentiation of immune cells, stimulating the leukocytes to the infection sites and promoting the proliferation of hematopoietic progenitor cells after viral infection. We also found that the serum level of IL6 was tightly associated with the biomarker cTnI in all patients with COVID-19 at different time points, which supports the point of view that the inflammation starting in and propagating from the lung or other initial organ injuries probably resulted in some bystander effects on other organs, such as the heart, due to amplifying inflammatory responses [9, 12].

Dysfunction of pulmonary ventilation, hypoxemia and other related symptoms in COVID-19 patients were indicators for disease progression to severe conditions or death in previous studies [13–15]. In some critical cases, patients with respiratory failure might develop ARDS, sepsis, multiorgan dysfunction or even septic shock [6]. In our study, the respiratory rate, chest distress and dyspnea were more frequent in severe patients. However, all the clinical symptoms related to pulmonary ventilation dysfunction and hypoxemia showed no difference no matter COVID-19 patients with or without cardiac injury. These findings were consistent with previous data, although these conclusions were not given enough attention in their article [8]. In addition, the PaO_2_ on admission and the P/F ratio after oxygen inhalation were not improved in cardiac damaged patients. Moreover, the biomarkers cTnI and BNP showed no correlation with these hypoxemia-related indicators. Therefore, we concluded that pulmonary ventilation dysfunction and oxygen inhalation insufficiency were not direct causes of cardiac injury or myocardial ischemia in patients with COVID-19.

Although the PSI, CURB65, APACHEII and SOFA scores were significantly higher in both severe patients and patients with cardiac damage, the pre-existing hypertension and SOFA scores on admission to the hospital in patients with COVID-19 were independent risk factors for patients progressing to cardiac injury (Table 8). Gu J and his colleagues had pointed out that immune and lung damage were the key features of coronavirus infection, accompanied by lymphocytopenia and inflammatory cytokine storms, which led to multiple organ infections or injuries, including in respiratory epithelial cells, the intestinal mucosa, the renal distal tubule epithelium and cerebral neurons [16, 17]. Human coronaviruses (SARS-CoV and SARS-CoV-2) target epithelial cells or immune cells by binding their spike protein to angiotensin-converting enzyme 2 (ACE2), which is expressed by epithelial cells of the lung, intestine, kidney, heart and blood vessels, thus priming the serine protease TMPRSS2 for S protein [18, 19]. Single-cell RNAseq data analysis of receptor ACE2 expression demonstrated that more than 7.5% myocardial cells had positive ACE2 expression, indicating that the heart or vessels could be directly infected and damaged in overloaded SARS-CoV-2 patients [20]. As patients with hypertension or diabetes were commonly treated with ACE inhibitors and angiotensin II type-I receptor blockers, an upregulation in the expression of ACE2 was mentioned in previous studies [18, 21, 22], which perhaps led to the heart being more vulnerable to SARS-CoV-2 infection. In our current study, pre-existing hypertension was observed in a significantly higher proportion in cardiac damaged patients, and it was an independent risk factor for COVID-19 patients developing myocardial damage in our multivariate logistic regression model. Furthermore, COVID-19 patients with cardiac injury were more likely to die in our study. However, some researchers pointed out that no difference in ACE2 expression or activity was found after antihypertensive calcium channel blocker treatment [23]. A study including 50 hospitalized hypertensive patients with laboratory-confirmed COVID-19 in Wuhan revealed no obvious difference in clinical characteristics between RAS blockers and non-RAS blockers groups [24]. Therefore, we inferred that SARS-CoV-2 might invade vessels by some uncertain pathways to reach the heart and damage myocardial cells by binding to upregulated ACE2, directly to lead to cardiac injury.

**Table 8:** Multivariate logistic regression analysis on the risk factors associated with cardiac injury in COVID-19 patients.

	**Univariable OR (95% CI)**	**P value**	**Multivariable OR (95% CI)**	**P value**
Demographics and clinical characteristics				
Age	1.08 (1.02-1.13)	0.004	1.07 (0.99-1.15)	0.087
Hypertension	4.00 (1.40-11.45)	0.010	3.28 (1.02-10.61)	0.047
Heart rate	1.03 (1.00-1.05)	0.032	--	--
Systolic pressure	1.05 (1.01-1.08)	0.004	--	--
PSI score	1.03 (1.01-1.05)	0.005	0.99 (0.95-1.03)	0.701
CURB65	2.73 (1.38-5.42)	0.004	1.43 (0.42-4.84)	0.568
APACHEII score	1.23 (1.09-1.40)	0.001	--	--
SOFA score	1.68 (1.20-2.33)	0.002	1.66 (1.10-2.52)	0.018
Laboratory parameters				
Lymphocytes	0.10 (0.02-0.50)	0.005	--	--
CRP	1.012 (1.00-1.02)	0.008	--	--
TB	1.11 (1.03-1.20)	0.005	--	--
Potassium	0.26 (0.09-0.72)	0.009	--	--
Calcium	0.92 (0.83-1.03)	0.126	--	--
IL2	1.18 (0.97-1.42)	0.099	--	--
IL6	1.01 (1.00-1.02)	0.161	--	--
LDH	1.01 (1.00-1.01)	0.001	--	--
BNP	1.00 (1.00-1.00)	0.003	--	--
CK	1.01 (1.00-1.01)	0.031	--	--
CKMB	1.04 (1.00-1.07)	0.029	--	--
D-dimer	1.02 (1.00-1.04)	0.100	--	--
P/F ratio	1.00 (1.00-1.01)	0.880	--	--

p values were calculated by multivariate logistic regression analysis. PSI score, pneumonia severity index score; CURB 65 score, Confusion/Urea/Respiratory rate/Blood pressure 65; APACHE-II score, Acute Physiology and Chronic Health Evaluation II score; SOFA score, Sequential Organ Failure Assessment. CRP, C-reactive protein; TB, total bilirubin; LDH, lactate dehydrogenase; BNP, brain natriuretic peptide; CK, creatine kinase; CKMB, creatine kinase-MB.

Our study has several inevitable limitations. First, the number of patients with cardiac injury and mortality was not very high in our study, but the proportions both were in reasonable ranges. Furthermore, the drugs that the patients with preexisting hypertension took before SARS-CoV-2 infection were not analyzed in our study due to the incomplete medical history collected from the patients.

## CONCLUSIONS

In conclusion, our study indicated that cardiac injury was an important indicator for patients with severe or fatal disease, and patients with preexisting hypertension and higher SOFA scores upon admission were more likely to progress to cardiac injury. Nevertheless, pulmonary ventilation dysfunction and oxygen inhalation insufficiency were not the main causes of cardiac injury in patients with COVID-19.

## MATERIALS AND METHODS

### Study participants

This retrospective cohort study included adult patients (≥18 years old) admitted to Renmin Hospital of Wuhan University from February 16 to March 21, 2020. All inpatients were confirmed to have COVID-19 by SARS-CoV-2 RNA detection. As this study focused on patients with or without cardiac injury, participants with pre-existing coronary heart disease or other myocardial diseases were excluded. This study was approved by the Ethics Committee of Renmin Hospital of Wuhan University.

### Data collection

Epidemiological data, demographic data, laboratory indicators, treatment details and outcome data were collected from electronic medical records and confidentially protected by assigning a deidentified ID to each patient. Laboratory indicators and treatment details were collected for at least 14 days on days 1, 3, 7 and 14. The degree of severity of COVID-19 patients (severe vs. non-severe) was defined at the time of admission, according to the American Thoracic Society guidelines for community-acquired pneumonia [25]. Cardiac injury was defined by cardiac biomarker (cardiac troponin I, [cTnI]) levels in the blood above the 99^th^-percentile of the upper reference limit. Patients with unavailable key information were excluded from our study.

### Statistical analysis

Categorical variables are presented as N, % and were compared using Fisher’s exact test or χ2 test. Continuous variables are presented as the mean ± SEM or median (interquartile range [IQR]) values and were compared using Student’s *t* test or the Mann-Whitney U test, as appropriate. To explore the risk factors associated with cardiac injury in patients with COVID-19, univariable and multivariate logistic regression models were used. Correlation analysis between the two parameters was performed using the Pearson correlation coefficient. Data were analyzed using SPSS version 22.0 (IBM), and statistical charts were generated using Prism 7.0 (GraphPad 7.0). For all the statistical analyses, *P* < 0.05 was considered significant.
